# Utilization and in‐hospital complications of catheter ablation for atrial fibrillation in patients with obesity and morbid obesity

**DOI:** 10.1002/clc.23795

**Published:** 2022-02-16

**Authors:** Narut Prasitlumkum, Ronpichai Chokesuwattanaskul, Wisit Kaewput, Charat Thongprayoon, Tarun Bathini, Boonphiphop Boonpheng, Saraschandra Vallabhajosyula, Wisit Cheungpasitporn, Krit Jongnarangsin

**Affiliations:** ^1^ Department of Medicine University of California Riverside Riverside California USA; ^2^ Department of Medicine, Division of Cardiovascular Medicine, King Chulalongkorn Memorial Hospital, Faculty of Medicine Chulalongkorn University Bangkok Thailand; ^3^ Department of Medicine, Faculty of Medicine Center of Excellence in Arrhythmia Research Chulalongkorn University Bangkok Thailand; ^4^ Department of Medicine, Department of Military and Community Medicine, Division of Nephrology Phramongkutklao College of Medicine Bangkok Thailand; ^5^ Department of Medicine Mayo Clinic Rochester Minnesota USA; ^6^ Department of Cardiology Bassett Medical Center Cooperstown New York USA; ^7^ Department of Medicine, David Geffen School of Medicine University of California Los Angeles California USA; ^8^ Department of Medicine, Section of Cardiovascular Medicine Wake Forest University School of Medicine Winston‐Salem North Carolina USA; ^9^ Division of Cardiac Electrophysiology University of Michigan Health Care Ann Arbor Michigan USA

**Keywords:** ablation, atrial fibrillation, complications, obesity

## Abstract

**Background:**

Real‐world data on atrial fibrillation (AF) ablation outcomes in obese populations have remained scarce, especially the relationship between obesity and in‐hospital AF ablation outcome.

**Hypothesis:**

Obesity is associated with higher complication rates and higher admission trend for AF ablation.

**Methods:**

We drew data from the US National Inpatient Sample to identify patients who underwent AF ablation between 2005 and 2018. Sociodemographic and patients' characteristics data were collected, and the trend, incidence of catheter ablation complications and mortality were analyzed, and further stratified by obesity classification.

**Results:**

A total of 153 429 patients who were hospitalized for AF ablation were estimated. Among these, 11 876 obese patients (95% confidence interval [CI]: 11 422–12 330) and 10 635 morbid obese patients (95% CI: 10 200–11 069) were observed. There was a substantial uptrend admission, up to fivefold, for AF ablation in all obese patients from 2005 to 2018 (*p* < .001). Morbidly obese patients were statistically younger, while coexisting comorbidities were substantially higher than both obese and nonobese patients (*p* < .01) Both obesity and morbid obesity were significantly associated with an increased risk of total bleeding, and vascular complications (*p* < .05). Only morbid obesity was significantly associated with an increased risk of ablation‐related complications, total infection, and pulmonary complications (*p* < .01). No difference in‐hospital mortality was observed among obese, morbidly obese, and nonobese patients.

**Conclusion:**

Our study observed an uptrend in the admission of obese patients undergoing AF ablation from 2005 through 2018. Obesity was associated with higher ablation‐related complications, particularly those who were morbidly obese.

**Abbreviations**
AF: atrial fibrillation
BMI: body mass index
CI: confidence interval
EP: electrophysiology
GA: general anesthesia
LA: left atrium
OR: odds ratio
RS: respiratory
UTI: urinary tract infection

## 1 INTRODUCTION

Obesity is a well‐established risk factor of both new‐onset and recurrent atrial fibrillation (AF).^1^ In obese patients, Several mechanisms promote AF, not only coexisting comorbidities but also inflammatory processes and atrial electrophysiological remodeling. For these reasons, American College of Cardiology/American Heart Association guidelines have recommended weight loss by comprehensive weight management programs to mitigate AF burdens and severities.^2^, ^3^ On the contrary, several studies have pointed out the obesity paradox effect owing to the favorable cardiovascular outcomes observed on anticoagulated AF patients with obesity compared to nonobese.^4^ Furthermore, one study suggested weight loss in obese patients who underwent AF ablation, surprisingly, did not improve AF recurrence rates.^5^

Catheter ablation has been widely accepted as a principal treatment for AF^6^ and has become the first line in particular subgroups such as younger and athletic patients.^7^ The majority of ablation outcomes in large clinical trials have come from exclusively high‐volume academic centers with experienced operators. As AF catheter ablation has been performed broadly, the results can be varying across regions depending on center experiences and practices. Real‐world data on AF ablation outcomes in obese populations have so far been scarce, especially the relationship between obesity and in‐hospital AF ablation trend and outcomes.

Thus, we evaluated the most recent admission trend and the impact of obesity in patients undergoing AF ablation on the hospital outcomes by utilizing the National Inpatient Sample (NIS) database, the current largest inpatient database from US inpatient settings.

## 2 METHODS

### 2.1 Data source

The National (Nationwide) Inpatient Sample is the largest all‐payer database of hospital inpatient stays in the United States. NIS contains discharge data from a 20% stratified sample of community hospitals and is a part of the Healthcare Quality and Utilization Project (HCUP), sponsored by the Agency for Healthcare Research and Quality (Introduction to the HCUP Nationwide Inpatient Sample 2009. http://www.hcup-us.ahrq.gov/db/nation/nis/NIS_2009_INTRODUCTION.pdf. Accessed January 18, 2015). Information regarding each discharge includes patient demographics, primary payer, hospital characteristics, principal diagnosis, up to 24 secondary diagnoses, and procedural diagnoses. The HCUP‐NIS does not capture individual patients but captures all information for a given admission. Institutional Review Board approval was not needed as this database was publicly available and deidentified. These data are available to other authors via the HCUP‐NIS database with the Agency for Healthcare Research and Quality.

### 2.2 Study patients and variables

International Classification of Diseases, Ninth Revision, Clinical Modification (ICD‐9‐CM) was used for reporting diagnoses and procedures in the NIS database during the study period. For each index hospitalization, the database provides a principal discharge diagnosis, additional diagnoses, and procedures, in which total maximum numbers vary per year. We identified patients 18 years of age or older with a primary diagnosis of AF (ICD‐9‐CM 427.31 and ICD‐10CM I48.0, I48.1, I48.2, I48.91) and underwent a CA procedure (ICD‐ 9‐CM 37.34 and ICD‐10CM 02583ZZ) from 2005 to 2018. To avoid selection bias and choose only the patient who had an ablation for AF, we have excluded all the patients with other arrhythmias or potential reasons for an ablation like atrial flutter (ICD 9CM 427.32 and ICD‐10CM I48.3, I48.4, I48.92), supraventricular tachycardia (ICD 9CM 427.0, 427.89, 426.7, 426.89 and ICD‐10CM I471), ventricular tachycardia (ICD‐9CM 427.1 and ICD‐10CM I472), Wolff‐Parkinson‐White syndrome/pre‐excitation syndrome (ICD‐9CM 426.7 and ICD‐10CM I456), “other premature beats” (ICD‐9CM 427.69 and ICD‐10CM I49.4, I49.40, I49.9), and cardiac dysrhythmia (ICD‐9CM 427.89 and ICD‐10CM I49.9). Furthermore, we excluded patients with either of the following cardiac procedures during the index hospitalization to avoid attributing their complications to the ablation procedure; (1) pacemaker implantation (00.50, 00.52, 00.53, 37.71–37.79, and ICD‐10CM 4B02XSZ) or (2) implantable cardioverter‐defibrillator insertion (37.94–37.98, 00.51, 00.54, and ICD‐10CM 4B02XTZ).

To determine the impact of obesity in patients undergoing AF ablation, body mass index (BMI) was used to stratify patients into nonobese (<30 kg/m^2^), obese (≥30 and ≤40 kg/m^2^), and morbidly obese (>40 kg/m^2^).^8^ In brief, The BMI is a measure of body fat based on individual (both men and women) height and weight. It is calculated as a person's weight in kilograms (kg) divided by their height in meters squared (m^2^). For the purpose of our analysis, we have identified the following ICD‐9‐CM/10CM codes for nonobesity (V85.0–V85.1, V85.21–V85.25), obesity (278.00, V85.3/E66.09, E66.8, E66.9, Z68.3×), and morbid obesity (278.01, V85.4×/E66.9, Z684×).

The following patient demographics were collected from the database, including age, sex, and race. In addition, associated comorbidities were identified by measures from the Agency for Healthcare Research and Quality. For the purposes of calculating Deyo‐Charlson Comorbidity Index (Deyo‐CCI), an additional list of comorbidities was identified from the database using ICD‐9‐CM codes and ICD‐10‐CM codes (Table S1). Deyo‐CCI is a modification of the CCI, containing 17 comorbid conditions. Higher Deyo‐CCI indicates a more severe condition and is an indicator of patient mortality 1 year after an admission.

### 2.3 Outcomes

We identified the common in‐hospital complications of CAs using the ICD‐9‐CM and ICD‐10‐CM diagnosis, and procedure codes using the same methodology as described in previous publications.^9^, ^10^, ^11^ These complications include (1) cardiac complications (postoperative cardiac block, myocardial infarction, cardiac arrest, congestive heart failure, and others); (2) pericardial complications (tamponade, hemopericardium, pericarditis, and pericardiocentesis); (3) vascular complications (arteriovenous fistula, blood vessel injury, accidental puncture, injury to the retroperitoneum, vascular complications requiring surgery, and other iatrogenic vascular complications); (4) postoperative hemorrhage or hematoma (including postoperative hemorrhage requiring blood transfusion); (5) postoperative stroke/transient ischemic attack; (6) pulmonary complications (pneumothorax/hemothorax, diaphragm paralysis, and postoperative respiratory [RS] failure); and (7) in‐hospital deaths.

In addition, we explored (8) gastrointestinal (GI) complications (esophagitis, esophageal ulcer, esophageal stricture, esophageal perforation, gastroesophageal laceration‐hemorrhage syndrome, burnt esophageal, and gastroparesis) and (9) skin complications (hair loss, erythema, burn, pressure ulcer, and radiation‐induced skin injury). Owing to Cluckey et al.,^12^ up to 2.9% of patients undergoing AF ablation were complicated by genitourinary infection, underlining its commonplace of this entity. For this reason, we included urinary tract infection (UTI) along with fever, septicemia, UTI, and postprocedural aspiration pneumonia, under infection complication (10) category. All codes used in identifying complications are summarized in Table S2. Readmissions for any reasons and outcomes which occurred after discharge were unavailable for the analysis as these data were not included in the NIS data set package.

### 2.4 Statistical analysis

Trend weight files provided by Agency for Healthcare Research and Quality were used to reflect national estimates. The *χ* ^2^ test and analysis of variance were used to compare categorical and continuous variables, respectively. Trends for continuous variables were tested using the nonparametric test for trend by Cuzick.^13^ In compliance with the data use agreement of HCUP nationwide databases, it was recommended to avoid reporting small numbers of observations (≤10) to minimize risks of person identifications.

To account for hospital‐level clustering of discharges, we generated a two‐level mixed‐effects logistic regression model to identify independent predictors of complications.^9^, ^10^ Congruent with Healthcare Cost and Utilization Project NIS design, hospital identification number was employed as a random effect with patient‐level factors clustered within hospital‐level factors. Candidate variables included patient‐level characteristics, Deyo‐CCI, and hospital‐level factors. For all analyses, we used both SPSS v24.0 (IBM Corp.) and STATA version 16. A *p* value less than .05 was considered significant.

## 3 RESULTS

### 3.1 AF ablation hospitalization among obese patients and demographic and characteristics profiles

Applying our inclusion and exclusion criteria, a total of 153 429 patients who were hospitalized for AF ablation were estimated. Among these, 11 876 obese patients (95% confidence interval [CI]: 11 422–12 330) and 10 635 morbid obese patients (95% CI: 10 200–11 069) were approximated. The majority of patients were male, White, Deyo‐CCI ≥ 1, with Medicaid or Private as a primary payer. Hypertension, congestive heart failure, diabetes, and chronic pulmonary disease were the most prevalent comorbidities. AF ablation was mostly performed in teaching hospitals (Table 1).

**Table 1:** Patient characteristics

	Nonobese	Obesity	Morbid obesity	*p* Value^a^
Unweighted numbers of participants^b^	26 913	2440	2150	N/A
Estimated numbers of participants^c^	130 962	11 832	10 635	
Mean age in years at admission^d^	65 ± 12	61 ± 10	62 ± 11	<.001
General demographic data^e^				
Indicator of sex				
Male	62.4%	60.6%	56.4%	<.001
Female	37.6%	39.4%	43.6%	
Primary expected payer				
Medicare	52.0%	40.1%	46.6%	<.001
Medicaid	2.7%	4.9%	5.7%	
Private insurance	42.3%	50.9%	43.8%	
Self‐pay	0.8%	1.2%	1.1%	
No charge	0.1%	0.3%	0.2%	
Other	2.2%	2.6%	2.6%	
Race				
White	88.4%	88.4%	85.5%	<.001
Black	3.4%	4.4%	6.8%	
Hispanic	3.9%	4.3%	5.1%	
Asian and Pacific islander	1.4%	0.5%	0.5%	
Native American	0.5%	0.5%	0.2%	
Other	2.4%	1.8%	1.9%	
Hospital region				
Northeast	36.5%	36.4%	27.3%	<.001
Midwest	39.4%	39.7%	29.6%	
South	17.4%	16.9%	34.0%	
West	6.7%	7.0%	9.1%	
Type of hospitals				
Government or private	43.8%	42.6%	15.6%	<.001
Government, nonfederal	3.9%	4.0%	6.2%	
Private, not‐for‐profit	42.2%	44.1%	65.8%	
Private, investor‐owned	9.8%	8.7%	12.0%	
Private, either not‐for‐profit or investor‐owned	0.3%	0.6%	0.3%	
Location/teaching				
Rural	1.9%	3.0%	2.8%	<.001
Urban nonteaching	23.5%	22.4%	17.3%	
Urban teaching	74.6%	74.6%	79.9%	
Bed sizes				
Small	6.6%	6.0%	8.9%	<.001
Medium	19.2%	20.6%	23.7%	
Large	74.2%	73.3%	67.4%	
Median household income				
First quartile	19.0%	20.6%	24.0%	<.001
Second quartile	23.4%	24.8%	26.1%	
Third quartile	26.8%	27.4%	26.2%	
Fourth quartile	30.9%	27.2%	23.8%	
Comorbidities and characteristics^e^				
Coronary artery disease	23.4%	23.4%	29.9%	<.001
Use of anticoagulants	30.9%	34.8%	46.0%	<.001
Coagulopathy	0.9%	0.9%	2.6%	<.001
Congestive heart failure	21.7%	23.9%	38.2%	<.001
Chronic pulmonary disease	14.5%	19.3%	25.7%	<.001
Peripheral vascular disease	4.1%	3.1%	7.5%	<.001
Any renal disease	5.2%	4.9%	10.6%	<.001
Hypertension	51.3%	69.7%	57.8%	<.001
Diabetes	15.4%	29.7%	35.3%	<.001
Length of stay^d^	2.6 ± 3.1	2.7 ± 3.0	3.5 ± 3.6	<.001

^a^ Estimated numbers of patients mean the weighted numbers of total sampled subjects who met the inclusion criteria described in the main manuscript. Its purpose is to provide an estimation of the total patients who were hospitalized in the represented year. For each NIS database package, trend weights factors, called “TRENDWT” for databases from 2012 up to 2018, and “DISCWT” for databases before 2012, were provided for a statistical estimation of single/multiyear analysis.

^b^ *p* < .05 considered statistical significance.

^c^ Unweighted numbers of patients mean the total number of sampled subjects who met the inclusion criteria described in the main manuscript. For each NIS database data set, it contains a discharge data from a 20% stratified sample of community hospitals and is a part of the Healthcare Quality and Utilization Project.

^d^ Continuous data; mean ± standard deviation; ANOVA for statistical comparison.

^e^ Categorical data; represented as percentages; *χ* ^2^ for statistical comparison.

Considering participants' profiles, all obese patients were statistically younger than nonobese patients (61 ± 10, 62 ± 11, 65 ± 12, for obese, morbidly obese, and nonobese patients respectively, *p* ≤ .001). Coexisting comorbidities were found significantly higher among all obese patients when compared to nonobese patients. The highest proportion of patients with Deyo‐CCI ≥ 2 was observed in morbidly obese patients, followed by obese and nonobese patients (*p* ≤ .001). The use of anticoagulation was highest in morbidly obese patients, followed by obese and nonobese patients (*p* ≤ .001; Table 1).

### 3.2 Trend in AF ablation among obese patients

**Figure 1:**
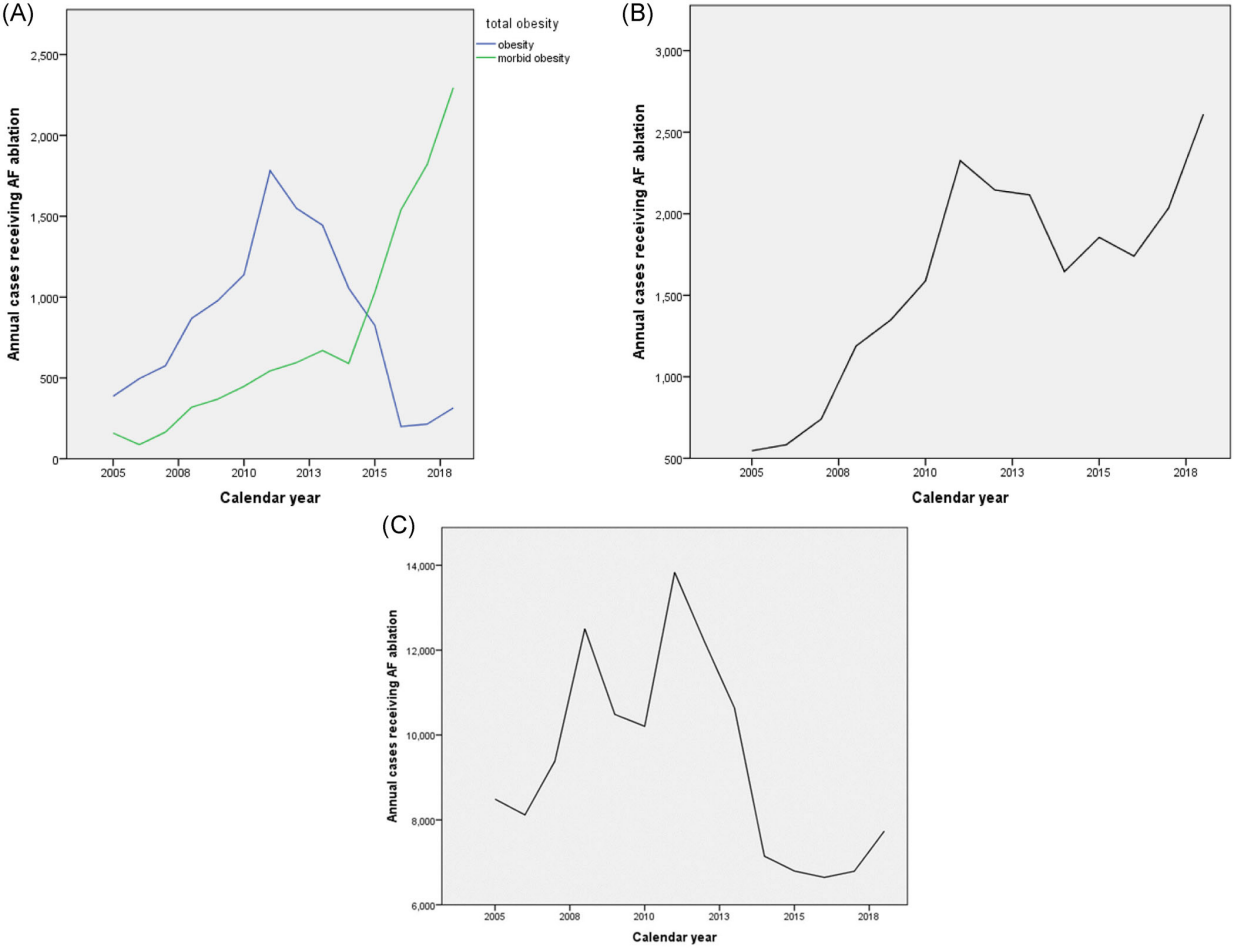
Annual trends of AF ablation admission in (A) obese and morbidly obese patients, (B) all obese patients, and (C) nonobese patients. AF, atrial fibrillation

According to our analysis, there was an uptrend admission for all obese patients undergoing AF ablation from 2005 to 2018, increasing from 2.4% to 11.4% (*p* < .001; Figure 1A). Essentially, the admission rates for AF ablation in morbidly obese patients were substantially rising from 1.5% to 21.3% (*p* < .001; Figure 1B). On the other hand, a significant downtrend was found from 2005 to 2018 among nonobese patients, potentially by a shift into an outpatient‐based procedure (*p* < .001), while a nonstatistical decrement in the admission trend of obese patients was observed (*p* = .114; Figure 1C).

### 3.3 Influence of obesity on in‐hospital complications and deaths

**Figure 2:**
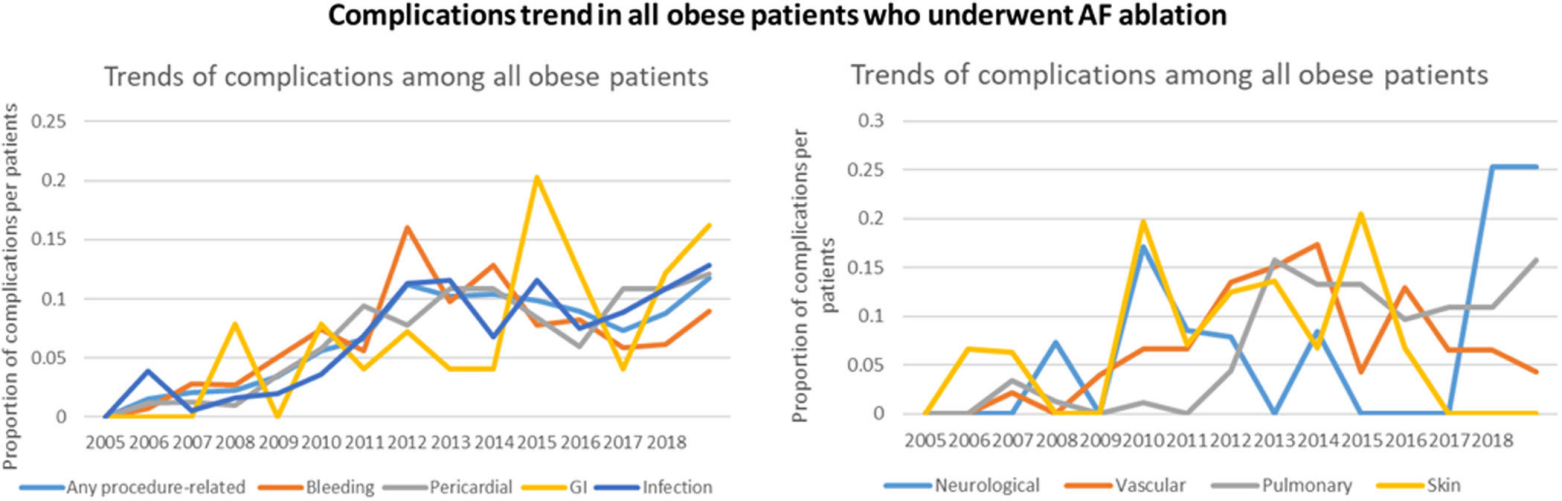
In‐hospital complications trend in all obese patients who underwent AF ablation. AF, atrial fibrillation

For ablation‐related complications, it was found 10.4% of the total cohort. Of these, total bleeding rates were the most common complication (4.9%), followed by total infection rates (2.8%) and total pericardial complications (1.7%). By obesity classifications, morbidly obese patients had the highest rates 14.3%, followed by obese patients 11.4% (*p* ≤ .001; Table 2). Moreover, from 2005 to 2018, there was a significant increment in ablation‐related complications trend in total obese patients, from 1.4% to 11.9% (*p* < .001). When considering each complication category, cardiac, pulmonary, pericardial, GI, and infection complications, were all statistically uptrend from 2005 to 2018 (*p* < .05; Figure 2 and Table S3).

**Table 2:** In‐hospital complications rates per each patients group

	Nonobese	*N*	Obesity	*N*	Morbid obesity	*N*	Total cohorts	*N*
Hemorrhagic complications	4.7%	6216	5.6%	663	5.8%	619	4.9%	7498
Pericardial complications	1.7%	2188	1.8%	212	1.9%	203	1.7%	2603
Vascular complications	0.5%	676	1.0%	114	1.1%	118	0.6%	908
Cardiac complications	0.9%	1179	0.8%	92	0.6%	59	0.9%	1330
GI complications	0.5%	651	0.7%	78	0.4%	45	0.5%	774
Pulmonary complications	0.8%	1032	1.2%	139	2.6%	273	0.9%	1444
Neurological complications	0.3%	356	0.1%	a	0.5%	49	0.3%	415
Infectious complications	2.7%	3517	2.7%	315	3.9%	419	2.8%	4251
Skin complications	0.3%	441	0.3%	39	0.3%	34	0.3%	514
Died during hospitalization	0.2%	315	0.2%	20	0.3%	35	0.2%	370
Any procedure‐related complications	10.3%	13 448	11.6%	1361	14.4%	1527	10.4%	16 336

^a^ No cell size < 11 was allowed to demonstrate per in compliance with the data use agreement of Healthcare Quality and Utilization Project nationwide databases.

To investigate the reasons for this observed trend, further trend analyses were obtained, which showed significant increases in mean age, higher proportion of coexisting comorbidities, including congestive heart failure, diabetes, renal failure, peripheral vascular disease, coagulopathy, and Deyo‐CCI (all *p* < .05; Tables S4 and S5).

**Figure 3:**
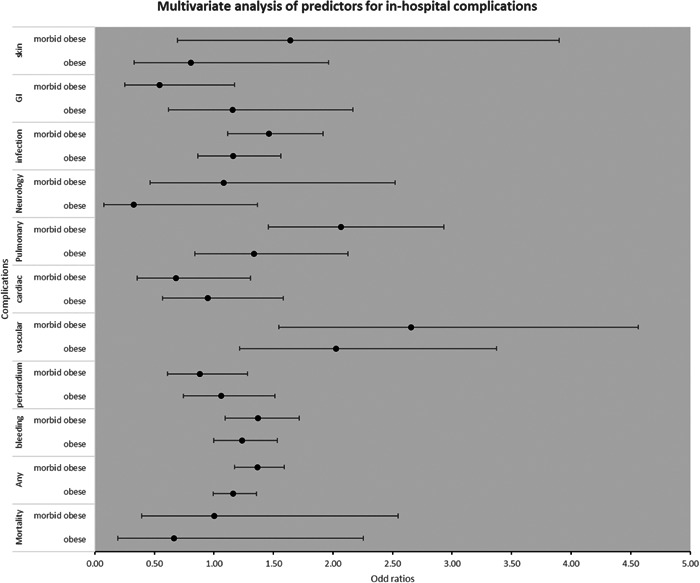
Multivariate analysis of predictors for in‐hospital complications

Further analyses were conducted to determine the influence of obesity on in‐hospital complications. Compared to nonobese patients, our study demonstrated that obesity was significantly associated with vascular complications (odds ratio [OR] 2.02, 95% CI: 1.21–3.37, *p* = .007). and bleeding complications (OR: 1.23, 95% CI: 0.99–1.54, *p* = .052). Marginally, it had an increased trend toward risks of ablation‐related complications (OR: 1.16, 95% CI: 1.00–1.36, *p* = .058). Similar results were noted in morbid obesity compared to nonobese patients, a statistical association with increased risks ablation‐related complications (OR: 1.36, 95% CI: 1.17–1.59, *p* < .001), bleeding (OR: 1.37, 95% CI: 1.09–1.72, *p* = .006) and vascular complications (OR: 2.65, 95% CI: 1.54–4.56, *p* < .001). Interestingly, only morbid obesity was substantially associated with higher total infection rates (OR: 1.89, 95% CI: 1.29–2.78, *p* = .025), pulmonary complications (OR: 2.07, 95% CI: 1.46–2.93, *p* < .001; Figure 3 and Table S5).

Of note, neither obesity nor morbid obesity was associated with increases in total cardiac complications, total GI complications, total vascular complications, total skin complications, total neurological complications, and total pericardial complications.

In‐hospital mortality rates in our cohort were extremely low, only 0.2%. There was no statistical difference in mortality rates between nonobese, obese, and morbidly obese patients (*p* > .05).

## 4 DISCUSSION

Using the largest all‐payer inpatient database in the United States databases from 2005 to 2018, recent admission trends and the impact of obesity on patients undergoing AF ablation were analyzed, especially safety aspects. First, there was a substantial uptrend admission rate, up to fivefold, for AF ablation in all obese patients from 2005 to 2018. Of these, AF ablation admission for morbidly obese patients was significantly increased Next, an increase in ablation‐related complications was noted from 1.4% to 11.9%. Of these, bleeding was the most common complication in all patients, followed by infection and pericardial complications. We found that both obesity and morbid obesity were associated with total vascular complications and bleeding events. In particular, only morbid obesity was statistically correlated with higher ablation‐related complications, infection, and pulmonary complications, while only a trend toward higher ablation related complications was found in obese patients Nevertheless, both obesity and morbid obesity were not associated with an increased risk of total cardiac, pericardial, GI, skin, and neurological complications. Finally, total hospital mortality rates were only 0.2% in total of our cohort, which was no different among obese, morbidly obese, and nonobese patients.

In prior studies,^14^, ^15^, ^16^, ^17^ the focus was to assess the impact of obesity on AF recurrence rates after the ablation. Cha et al.^17^ demonstrated that AF ablation had a satisfactory performance on obese patients, with freedom of AF recurrence up to 70%–72% in 12 months follow‐up and improved their quality of life. On the contrary, the remainders^14^, ^15^, ^16^ suggested obesity neither was independent nor reduced the ablation efficacy. Nevertheless, all these studies did not extensively examine the obesity influence on complications aspects. On the other hand, our scope was to provide recent trends in admission for AF ablation and the implication of obesity onto hospital courses, essentially complications and death rates, by utilizing the pooled data of obese patients who underwent AF ablation in the inpatient setting. Our analysis found an increasing trajectory in both admission rates in all obese patients and their complications rates from 2005 to 2018, predominated by morbidly obese patients. Overall, these findings are explained by higher mean age and comorbidities, and a rise of patients with higher Deyo‐CCI in later years, as shown in our result sections. Speculatively, sicker patients were more likely preselected for admission for safety purposes, particularly in morbid obesity patients. The assumption may explain the higher complications rates corresponding to the admission uptrend from 2005 to 2018. Despite this explanation, it is immature to disregard the influence of obesity on complications rates. Indeed, more pieces of evidence are required to implement this unsolved domain.

### 4.1 Obesity and its in‐hospital complications impact

Very few studies described the potential complication risks of being overweight. One study^18^ found morbid obesity had up to 3.1‐fold complication rates, and 5% per 1 unit increment in BMI, while the other^19^ only showed a modest increase in total complications. However, the preponderance of previous studies negated the influence of obesity on complications.^14^, ^15^, ^16^ Of these, all obese patients were gathered into one group as one variable, which may confound its true association. Also, complication rates were not the outcome of interest, precluding the complete investigation. Contrastingly, our objective was to resolve this conflicting data, explicating whether obesity portends any adverse outcomes in the setting of hospitalization for AF ablation. Conclusively, both obesity and morbid obesity were associated with higher complication rates compared to nonobese patients. This finding emphasizes the disadvantage of obesity, despite the “obesity paradox” previously described in anecdotal literature.^20^, ^21^

In obese patients with BMI 30–39, total complication rates were implicated by a substantial portion of vascular and bleeding complications. This finding was intriguingly opposite to a myriad of former studies which described favorable outcomes in the obese population.^22^, ^23^, ^24^ In those studies, thinner patients were older and frailer and had more comorbidities, of which all were associated with poorer outcomes. Moreover, the bleeding incidence was lower among patients with higher BMI undergoing cardiac intervention,^25^, ^26^ visibly dissonant to our result. Therefore, it has been proposed that obese patients tend to have larger vessel sizes, advantageously reducing bleeding risks and vascular insults. In contrast, a higher Charlson Deyo classification index, comorbidities, and, importantly, a larger proportion of females were observed in both obese and morbidly obese patients according to our database. Use of anticoagulation was also found higher in both obese and morbidly obese compared to nonobese patients. As a result, this may partly result in a greater extent in bleeding vulnerabilities in patients with pre‐existing unhealthy underlying conditions, despite appropriate indications. These may offset obesity's merits, exposing its more downsides.

Our analysis demonstrated a significant association between higher pulmonary complication risks and morbidly obese patients. This finding is consistent with several previous studies, highlighting the increased risk of postoperative RS failures among obese patients.^27^, ^28^, ^29^ Alterations in RS anatomy and physiology are notably changed, in patients with large body habitus. Limited lungs expansion due to mass loading leads to reduced breathing capacity,^30^ described as a restrictive lungs pattern. Furthermore, ineffective diaphragmatic function, V/Q mismatch, increased pulmonary pressures, and, importantly, sleep apnea, both central and obstructive, were well‐described in this population.^31^, ^32^, ^33^ As a result, alveolar hypoventilation ensues, culminating in a significant breathing compromise and a higher predisposition to lungs injury.

Essentially, most operators in the US prefer general anesthesia (GA) over left atrium (LA) for AF ablation,^34^ as it ensures LA geometry accuracy during electrophysiology study, provides comfortability for the sake of longer duration, and controls RS cycles to improve contact forces and minimize unwanted LA artifacts.^35^, ^36^ Despite this courtesy, the use of GA is totally not without concerns, particularly in overweight patients. Physiological changes from the GA process may compound their RS status by promoting atelectasis, worsening airway patency, and interfering with breathing in these patients.^37^ This interplay under pathological changes in obesity results in acute RS failure either in hypoxic, hypercapnia, or a combination form.

Of note, we found a statistical association between morbid obesity and infection complications. It is uncertain why our study failed to show an association between nonmorbid obesity and total infection rates. Uninvestigated and unknown residual biases from a retrospective design may come into play for this reason. Moreover, specifying obesity by BMI may incorrectly classify subjects with high lean but low‐fat mass into obese or nonobese groups. Owing to its poor sensitivity to detect obese patients defined by % body fat,^38^ BMI itself cannot be used to differentiate the contribution from lean mass. On the other hand, higher cut‐off BMI, especially ≥40, increases the likelihood of acquiring actual patients with significantly high‐fat composition. For this reason, morbid obesity, aka class III obesity, was alluded to be a strong predictor for in‐hospital infection events. Indirectly, many previous publications suggested obesity as a risk factor of hospital infection in postcoronary bypass surgery.^39^, ^40^ To the best of our knowledge, no prior studies were examined whether obesity was related to infection risks in cardiac intervention settings, aka PCI or catheter ablation. Our study is the first to show this paramount correlation.

### 4.2 Limitation

Our study has several limitations. First, as the NIS database provided only inpatient information, readmissions for any reason were unanalyzable owing to unprovided information. Also, residual biases from retrospective design cannot be excluded, despite a comprehensive covariate adjustment in our analysis. Second, owing to the same reason, this study may over/underestimate the true impact of obesity given the recent AF ablation trend in an outpatient setting. Furthermore, susceptibility to error coding is hardly evitable in administrative databases. Despite this limitation, this is the largest representative data of an inpatient AF ablation best reflecting real‐world experience. Third, as BMI was used to stratify obesity classifications, it may not truly represent adiposity in certain populations, particularly the younger population, which correlated more to lean body/muscle mass.^41^ Obese status in the current databases may miscoded given this possible issue. Fourth, some factors which may affect the outcomes, for example, medications, especially types of anticoagulation, procedural techniques, fluoroscopy time, and use of hemostasis device, are not provided by NIS. Fifth, delayed onset complications, such as pulmonary vein stenosis and atrioesophageal fistula, cannot be accurately assessed in this study as these complications tend to occur after discharges. Moreover, the efficacy of AF ablation was unable to be determined owing to no available ICD codes for this aspect.

## 5 CONCLUSION

Based on the largest all‐payer inpatient database in the United States, our study observed an increase in trends of complications in obese patients undergoing AF ablation. Moreover, obesity was associated with higher ablation‐related complications, particularly those who were considered morbidly obese.

## AUTHOR CONTRIBUTIONS

*Study design, literature review, statistical analysis, manuscript revision, intellectual revisions, and mentorship*: Narut Prasitlumkum, Wisit Cheungpasitporn, Wisit Kaewput, Ronpichai Chokesuwattanaskul, Krit Jongnarangsin. *Data management, data analysis, drafting manuscript*: Tarun Bathini, Charat Thongprayoon, Ronpichai Chokesuwattanaskul, Saraschandra Vallabhajosyula, Krit Jongnarangsin. *Access to data*: Narut Prasitlumkum, Wisit Kaewput, Wisit Cheungpasitporn, Charat Thongprayoon, Tarun Bathini, Boonphiphop Boonpheng. *Final approval*: Narut Prasitlumkum, Wisit Cheungpasitporn, Wisit Kaewput, Charat Thongprayoon, Tarun Bathini, Boonphiphop Boonpheng, Saraschandra Vallabhajosyula, Ronpichai Chokesuwattanaskul, Krit Jongnarangsin. All authors had access to the data and a role in writing the manuscript.

## Supporting information

Supporting information.

Supporting information.

## Data Availability

The data for this systematic review and all potentially eligible studies are publicly available through the Open Science Framework (https://osf.io/p7d6v/).
