# Supplementary material for: Utilization and in‐hospital complications of catheter ablation for atrial fibrillation in patients with obesity and morbid obesity
Source: Clin Cardiol. 2022 Feb 16;45(4):407–16. doi: 10.1002/clc.23795 (PMC9019886; doi:10.1002/clc.23795)
Supplement: Supplementary file 1 — Supporting information. [file CLC-45-407-s002.docx]

**Supplementary file**

**Supplemental Table 1.** **Deyo Modification of Charlson Comorbidity Index**

| **ICD-9 CM codes** | **ICD-10 CM codes** | **Condition** | **Score** |
| --- | --- | --- | --- |
| 410 – 410.9 | I21, I22, I25.2 | Myocardial infarction | 1 |
| 428 – 428.9 | I50 | Congestive heart failure | 1 |
| 433.9, 441 – 441.9, 785.4, V43.4 | I70, I71, Z95 | Peripheral vascular disease | 1 |
| 430 – 438 | I60, I61, I62, I63, I65, I66, I67, I69, G45 | Cerebrovascular disease | 1 |
| 290 – 290.9 | F01, F02, F03, G30 | Dementia | 1 |
| 490 – 496, 500 – 505, 506.4 | J40, J41, J42, J44, J43, J45, J46, J47, J67, J44, J60, J61,J62, J63, J66, J64, J65 | Chronic pulmonary disease | 1 |
| 710.0, 710.1, 710.4, 714.0 – 714.2, 714.81, 725 | M32, M33, M34  M05, M060 | Rheumatologic disease | 1 |
| 531 – 534.9 | K25, K26, K27, K28 | Peptic ulcer disease | 1 |
| 571.2, 571.5, 571.6, 571.4 – 571.49 | K70, K73, K74 | Mild liver disease | 1 |
| 250 – 250.3, 250.7 | E10.9, E11.9, E13.9, E14.9, E10.1, E10.10, E10.11, E11.1, E11.10, E11.11, E13.1, E13.11, E13.10, E14.1 | Diabetes | 1 |
| 250.4 – 250.6 | E10.2 E10.21 E10.22 E10.29 E11.2 E11.21 E11.22 E11.29 E13.2 E13.21 E13.22 E13.29 E14.2 E14.3 E14.4 E10.4  E10.40 E10.41 E10.42 E10.43 E10.44 E10.49 E13.4 E13.40 E13.41 E13.42 E13.43 E134.4 E13.49 E11.4 E11.40 E11.41  E11.42 E11.43 E11.44 E11.49 | Diabetes with chronic complications | 2 |
| 344.1, 342 – 342.9 | G81, G82, G41.0 | Hemiplegia or paraplegia | 2 |
| 582 – 582.9, 583 – 583.7, 585, 586, 588 – 588.9 | N03, N052, N053, N054, N055, N056, N072, N073, N074, N01,N18, N19, N25 | Renal disease | 2 |
| 140-172.9, 174-195.8, 200-208.9 | C0, C1, C2, C3, C40, C41, C43, C45, C46, C47, C48, C49, C5,  C6, C70, C71, C72, C73, C74, C75, C76, C80, C81, C82, C83,  C84, C85, C883, C887, C889, C900, C901, C91, C92,  C93, C940, C941, C942, C943, C9451, C947,C95, C96 | Any malignancy including leukemia and lymphoma | 2 |
| 572.2 – 572.8 | K72.9 K72.90 K72.91 K76.6 K76.7 K72.1 K72.10 K72.11 | Moderate or severe liver disease | 3 |
| 196-199.1 | C77, C78, C79, C80 | Metastatic solid tumor | 6 |
| 042 – 044.9 | B20, B21, B22, B23, B24 | Acquired Immunodeficiency syndrome (AIDS) | 6 |

**Supplemental Table 2. ICD-9-CM and ICD-10-CM Codes Used for Analysis of Complications**

| **Complication** | **ICD-9-CM Code(s)** | **ICD-10-CM Code(s)** |
| --- | --- | --- |
| Pericardial | 423  423.3  37  420.9 | I31.2 I3.14 0W9D3ZZ 0W9D3ZX 0W9D30Z I3.09 I3.2 I30.0 I30.8 |
| Cardiac | 997.1 | I97.89 I97.710 I97.790 I97.88 |
| Pulmonary | 512.1-512.2, 511.8  519.4  518.51, 518.53  997.3 | J95.811 J95.812 J93.11 J93.12 J93.81 J93.82 J93.9 J98.6 J96.00 J95.821 J95.822 J96.20 J95.89 J95.88 J95.859 J95.851 |
| Hemorrhage/Hematoma | 998.11-998.12  285.1  (998.11-998.12, 285.1) AND (99.01-99.09) | I97.42 I97.610 I97.620 I97.630 I97.410 I97.42 D62 30243N0 30243H0 30233N0 30233H0 30233H1 30243H1 30243W0 30243H0 30233W0 30233N1 30233P1 30243N1 30243P1 30233R1 30243R1 30243W1 30243V1 30243T1 30233W1 30233V1 30233T1 30233J1 30233K1 301233L1 30233M1 30243J1 30243K1 30243L1 30243M1 3E033GC 3E043GC 3E053GC 3E063GC 30243Q1 30233Q1 |
| Vascular | 998.2, e8700-e709  900-904  447  8680.4  39.31, 39.41, 39.49, 39.52, 39.53, 39.56, 39.57, 39.58, 39.59, 39.79  997.2, 997.7 | I97.52 S09.0XXA S15, S25, S35, S36.899A, S45, S55.009A, S65, S75, S85, S95.109A, S95.8X-9X, T14.90XA I770  S36899A, 0W3, 02V-06V, 02U-06U, 02S-06S, 02C-06C, 02Q-06Q, |
| Infection | 780.60, 780.62  038.*, 995.91-995.92, 998.02, 790.7, 999.3*, 998.5*, 599.0  997.32  998.5*  999.3*  559.0 | A40.9 A41.2 A41.01 A41.02 A41.1  A40.3 A41.4 A41.50 A41.3 A41.51  A41.52 A41.53 A41.59 A41.89 A41.9  T80.219A T80.211A T80.212A T80.22XA T80.29XA  K68.11 N39.0 |
| Neurological | 997  997.01  997.02  435.9 | G97.81 G97.82 I97.821 G45.9 |
| GI complications | 53.0X, 94.72 | K20.9, K21.0, K20.8, K22.10, K22.11  K22.2, K22.3, K22.6, K22.8, K22.9 T28.1XXA |
| Skin complications | 704.09, 704.00, 695.9, 692.82, 707*, 942* | L65.8, L65.9, L53.9, L59.9, L59.8, L58.0, L58.9, L89*, T21* |

**Supplementary table 3.** **Complications trend in all obese patients who underwent AF ablation**

| **Year** | **Any procedure-related** | **Bleeding** | **Pericardial** | **GI** | **Infection** | **Cardiac** | **Neurological** | **Vascular** | **Pulmonary** | **Skin** |
| --- | --- | --- | --- | --- | --- | --- | --- | --- | --- | --- |
| 2005 | 1.5% | 0.7% | 1.2% | 0.0% | 3.9% | 3.4% | 0.0% | 0.0% | 0.0% | 6.7% |
| 2006 | 2.1% | 2.8% | 1.3% | 0.0% | 0.5% | 3.4% | 0.0% | 2.2% | 3.4% | 6.3% |
| 2007 | 2.3% | 2.7% | 1.0% | 7.9% | 1.6% | 0.0% | 7.3% | 0.0% | 1.3% | 0% |
| 2008 | 3.4% | 5.1% | 3.5% | 0.0% | 2.0% | 0.0% | 0.0% | 4.0% | 0.0% | 0% |
| 2009 | 5.6% | 7.4% | 5.8% | 7.9% | 3.6% | 2.9% | 17.1% | 6.7% | 1.1% | 19.7% |
| 2010 | 6.6% | 5.6% | 9.4% | 4.1% | 6.9% | 15.2% | 8.6% | 6.7% | 0.0% | 7.1% |
| 2011 | 11.2% | 16.0% | 7.8% | 7.2% | 11.3% | 8.9% | 7.9% | 13.5% | 4.4% | 12.5% |
| 2012 | 10.2% | 9.8% | 10.9% | 4.1% | 11.6% | 13.2% | 0.0% | 15.1% | 15.8% | 13.6% |
| 2013 | 10.4% | 12.9% | 10.9% | 4.1% | 6.8% | 16.6% | 8.4% | 17.3% | 13.3% | 6.8% |
| 2014 | 9.9% | 7.8% | 8.4% | 20.3% | 11.6% | 3.3% | 0.0% | 4.3% | 13.3% | 20.5% |
| 2015 | 9% | 8.2% | 6.0% | 12.2% | 7.5% | 9.9% | 0.0% | 13.0% | 9.7% | 6.8% |
| 2016 | 7.3% | 5.9% | 10.9% | 4.1% | 8.9% | 6.6% | 0.0% | 6.5% | 10.9% | 0% |
| 2017 | 8.8% | 6.2% | 10.9% | 12.2% | 10.9% | 6.6% | 25.3% | 6.5% | 10.9% | 0% |
| 2018 | 11.8% | 9.0% | 12.1% | 16.2% | 12.9% | 9.9% | 25.3% | 4.3% | 15.8% | 0% |
| P value | <0.001 | 0.346 | <0.001 | <0.001 | <0.001 | 0.01 | 0.126 | 0.986 | <0.001 | 0.966 |

**Supplementary table 4.** **Comorbidities trend in all obese patients who underwent AF ablation**

| **Year** | **Deyo Charlson comorbidity index** | | | **Diabetes** | **Congestive heart failure** | **Any renal disease** | **chronic pulmonary disease** | **Peripheral vascular disease** | **Coronary artery disease** | **Coagulopathy** | **Hypertension** |
| --- | --- | --- | --- | --- | --- | --- | --- | --- | --- | --- | --- |
|  | **0** | **1** | **≥2** |  |  |  |  |  |  |  |  |
| 2005 | 3.3% | 2.2% | 1.6% | 1.8% | 1.4% | 0.0% | 2.1% | 0.4% | 1.1% | 0.0% | 4.7% |
| 2006 | 4.2% | 2.6% | 1.0% | 1.6% | 1.4% | 0.3% | 1.7% | 0.4% | 2.1% | 1.3% | 5.1% |
| 2007 | 4.1% | 3.8% | 2.1% | 3.4% | 2.1% | 0.6% | 3.1% | 0.0% | 3.0% | 0.0% | 6.1% |
| 2008 | 8.0% | 5.7% | 2.2% | 4.3% | 2.7% | 0.0% | 3.3% | 2.1% | 3.9% | 1.3% | 9.2% |
| 2009 | 7.4% | 7.1% | 3.7% | 5.7% | 4.1% | 2.0% | 4.7% | 3.4% | 5.5% | 0.0% | 7.9% |
| 2010 | 8.0% | 7.1% | 5.9% | 7.5% | 5.3% | 4.0% | 5.7% | 4.2% | 5.1% | 5.1% | 8.2% |
| 2011 | 13.0% | 11.5% | 8.1% | 10.2% | 8.3% | 6.3% | 9.3% | 3.4% | 9.3% | 0.0% | 12.2% |
| 2012 | 10.0% | 10.2% | 7.6% | 9.9% | 7.3% | 5.2% | 8.8% | 5.1% | 8.6% | 1.3% | 10.3% |
| 2013 | 9.6% | 10.5% | 7.4% | 9.0% | 7.8% | 7.2% | 8.4% | 6.3% | 8.9% | 2.5% | 9.4% |
| 2014 | 6.3% | 6.5% | 8.9% | 8.9% | 8.5% | 8.1% | 7.8% | 5.1% | 7.4% | 0.0% | 6.3% |
| 2015 | 6.7% | 7.1% | 10.8% | 8.5% | 10.0% | 15.3% | 9.5% | 10.1% | 8.9% | 7.6% | 6.0% |
| 2016 | 5.7% | 6.8% | 10.7% | 7.9% | 11.0% | 12.7% | 9.7% | 17.3% | 9.8% | 30.4% | 5.7% |
| 2017 | 6.4% | 8.2% | 12.6% | 8.8% | 12.5% | 14.7% | 10.7% | 22.8% | 11.3% | 24.1% | 4.5% |
| 2018 | 7.3% | 10.6% | 17.3% | 12.5% | 17.7% | 23.6% | 15.2% | 19.4% | 15.2% | 26.6% | 4.4% |
| P value | < 0.001 | | | < 0.001 | < 0.001 | < 0.001 | < 0.001 | < 0.001 | < 0.001 | < 0.001 | 0.684 |

**Supplementary table 5. Multivariate analysis of predictors for in-hospital complications in patients who underwent an AF ablation between 2005 and 2018**

| **Complications** | **Category** | **OR** | **95% CI** | **P value** |
| --- | --- | --- | --- | --- |
| All reference is non-obesity | | | | |
| Any procedure-related complications | Obese | 1.16 | 1.00 - 1.36 | 0.058 |
|  | Morbidly obese | 1.37 | 1.17 – 1.59 | < 0.001 |
| Total infection events | Obese | 1.16 | 0.86 - 1.56 | 0.126 |
|  | Morbidly obese | 1.46 | 1.12 – 1.92 | 0.006 |
| Total bleeding | Obese | 1.24 | 1.00 - 1.53 | 0.052 |
|  | Morbidly obese | 1.77 | 1.09 – 1.72 | 0.006 |
| Total GI complications | Obese | 1.16 | 0.86 - 1.56 | 0.644 |
|  | Morbidly obese | 0.54 | 0.25 - 1.17 | 0.121 |
| Total vascular complications | Obese | 2.02 | 1.21 – 3.37 | 0.007 |
|  | Morbidly obese | 2.66 | 1.54 - 4.56 | <0.001 |
| Total pericardial complications | Obese | 1.06 | 0.74 - 1.51 | 0.745 |
|  | Morbidly obese | 0.88 | 0.61 - 1.28 | 0.512 |
| Total pulmonary complications | Obese | 1.34 | 0.84 – 2.12 | 0.22 |
|  | Morbidly obese | 3.92 | 2.29 - 6.71 | < 0.001 |
| Total neurological complications | Obese | 0.32 | 0.07 - 1.37 | 0.125 |
|  | Morbidly obese | 1.81 | 0.59 - 5.51 | 0.299 |
| Total cardiac complications | Obese | 0.89 | 0.52 - 1.52 | 0.677 |
|  | Morbidly obese | 0.54 | 0.19 - 1.50 | 0.235 |
| Total skin complications | Obese | 0.81 | 0.33-1.96 | 0.636 |
|  | Morbidly obese | 1.64 | 0.69-3.90 | 0.26 |
| In-hospital mortality | Obese | 0.66 | 0.19-2.25 | 0.511 |
|  | Morbidly obese | 1.00 | 0.39-2.55 | 0.997 |
