# Supplementary figures and images for: Utilization and in‐hospital complications of catheter ablation for atrial fibrillation in patients with obesity and morbid obesity
Source: Clin Cardiol. 2022 Feb 16;45(4):407–16. doi: 10.1002/clc.23795 (PMC9019886; doi:10.1002/clc.23795)

## Complications trend in all obese patients who underwent AF ablation

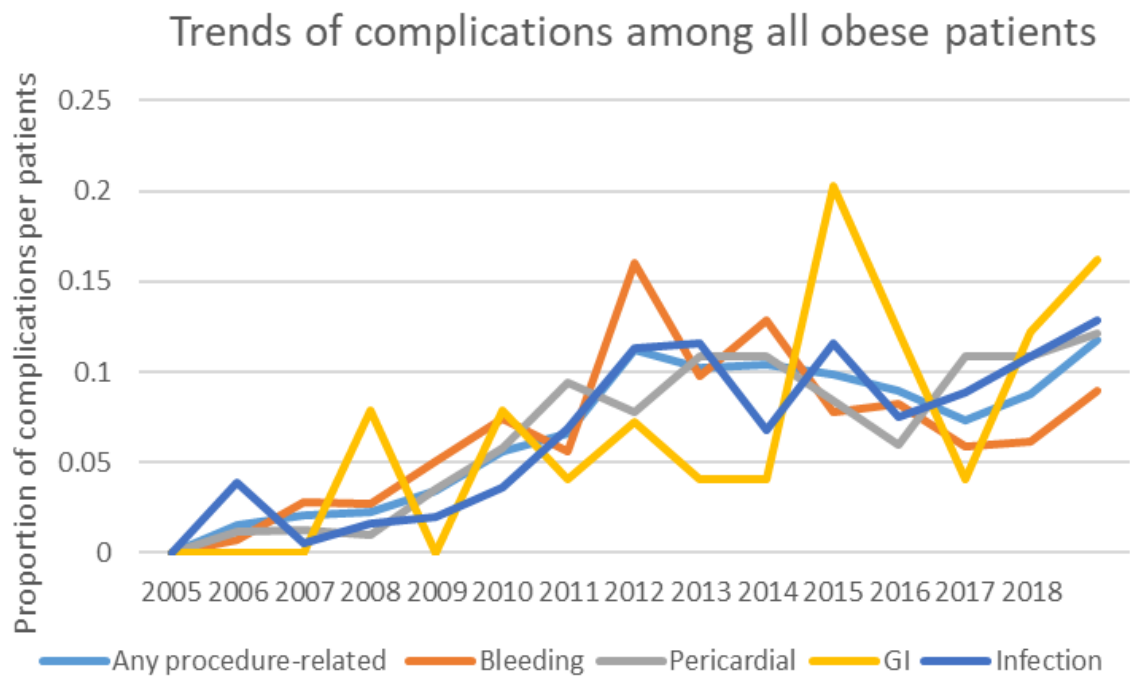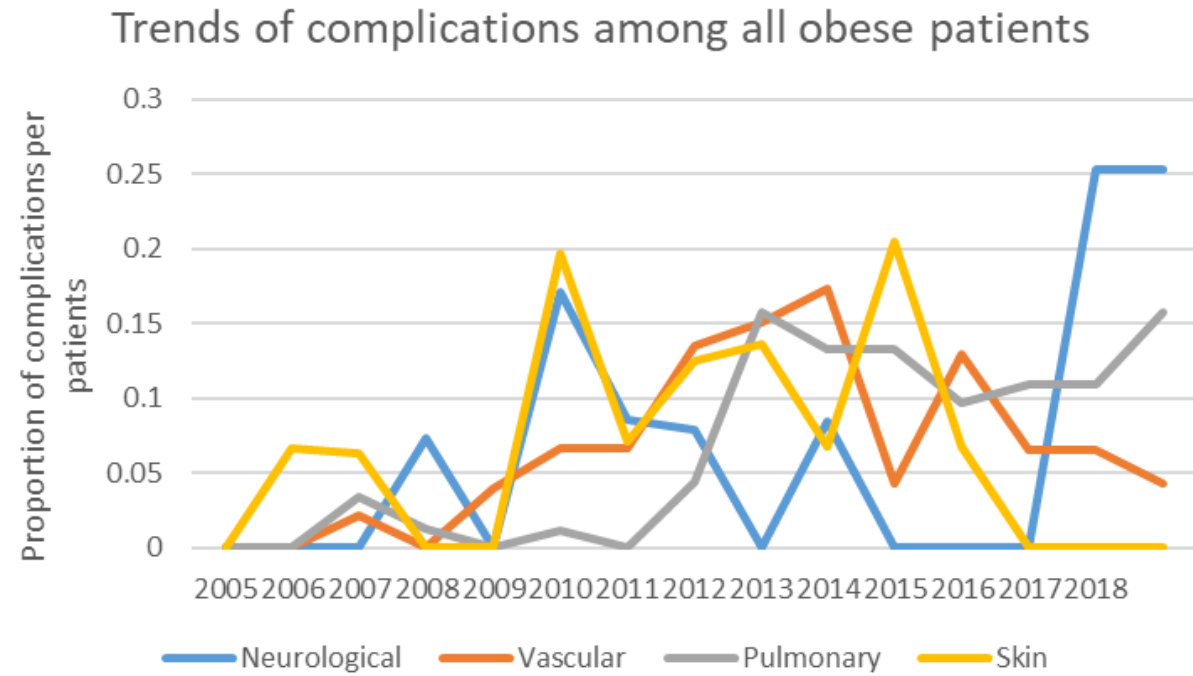

Supplement: Supplementary file 2 — Supporting information. [file CLC-45-407-s001.pdf]
